# Stable Vacancy-Rich Sodium Vanadate as a Cathode for High-Performance Aqueous Zinc-Ion Batteries

**DOI:** 10.3390/nano15120940

**Published:** 2025-06-17

**Authors:** Zhibo Xie, Yongru Qu, Fuwei Kong, Ruizheng Zhao, Xianfen Wang

**Affiliations:** 1 Institute of Materials for Energy and Environment, College of Materials Science and Engineering, Qingdao University, Qingdao 266071, China; xiezb202206@163.com (Z.X.); 13156951663@163.com (Y.Q.); 15328703051@163.com (F.K.); 2 Interdisciplinary Research Center for Sustainable Energy Science and Engineering (IRC4SE2), Engineering Research Center of Advanced Functional Material Manufacturing of Ministry of Education, School of Chemical Engineering, Zhengzhou University, Zhengzhou 450001, China

**Keywords:** zinc-ion batteries, vanadium-based materials, oxygen vacancies, cationic pre-embedding

## Abstract

Vanadium-based cathodes are promising for aqueous zinc-ion batteries (ZIBs) due to the large interlayer distance. However, the poor stability of electrode materials due to the dissolution effects has severely hindered the commercial development. To address this challenge, we propose an in situ NH_4_^+^ pre-intercalation strategy to enhance the electrochemical performance of Na_0.76_V_6_O_15_ (NaVO), thereby optimizing its structural stability and ionic conductivity. Moreover, NH_4_^+^ pre-intercalation introduced a large number of oxygen vacancies and defects into the material, causing the reduction of V^5+^ to V^4+^. This transformation suppresses the dissolution and enhances its conductivity, thereby significantly improving the electrochemical performance. This modified NaNVO cathodes deliver a higher capacity of 456 mAh g^−1^ at 0.1 A g^−1^, with a capacity retention of 88% after 140 cycles and a long lifespan, maintaining 99% of its initial capacity after 2300 cycles. This work provided a new way to optimize the cathode for aqueous zinc-ion batteries.

## 1. Introduction

Due to the increasing demand for sustainable energy, the development of low-cost energy storage technology has become a pressing issue. Although lithium-ion batteries (LIBs), which currently dominate the market, have superior energy density, there are major challenges in terms of scarce lithium resources and the high safety concerns of LIBs [1,2,3,4]. Aqueous zinc-ion batteries (ZIBs) have been proposed as an alternative candidate to LIBs due to the low cost and high safety in the specific region [2,5]. Cathodes with high energy density are of critical promise for the commercial future, such as Prussian blue analogs (PBAs) [6,7], manganese oxides [8,9,10], and vanadium-based compounds [11,12,13,14,15,16]. PBAs offer excellent cycling stability owing to their adjustable frameworks, but they suffer from limited capacity [17]. Manganese-based cathodes with low cost and high capacity have attracted intense attention [3]. Vanadium-based compounds with various coordination numbers and oxidation states offer high capacity and superior performance [18]. However, the poor cycling stability still exists and prevents them from further applications.

As a potential candidate cathode for ZIBs, V_2_O_5_ can be easily obtained with various synthesis methods [19,20,21,22]. However, the inherent challenge for this cathode is the narrow interlayer spacing of 4.3 Å, which not only impedes the Zn^2+^ transport but also results in structural collapse [23,24]. Increasing the interlayer distance in V_2_O_5_ is an effective consensus to address the poor cycling stability and low capacity [25]. Consequently, several intercalated oxides, such as LiV_2_O_5_·nH_2_O [26], Na_x_V_2_O_5_·nH_2_O [27], K_0.486_V_2_O_5_ [28], Ag_0.33_V_2_O_5_ [29], V_2_O_5_·nH_2_O [30], Zn_x_V_2_O_5_·nH_2_O [31], have been reported with improved performance. Nevertheless, the solubility issue of vanadium-based cathode materials at low current densities is still a crucial challenge to maintaining the stability of the cathodes. The side reactions with active water molecules and the simultaneous insertion of H^+^ and water molecules thereby aggravate the dissolution of the cathode electrodes. Therefore, mitigating the solubility issue of vanadium-based materials is essential in tackling this challenge for the vanadate cathodes.

NaVO is composed of distorted VO_6_ octahedra that share vertices and edges, thus forming a tunnel-like framework (Figure S1). The large channels within facilitate the rapid insertion and extraction of Zn^2+^. However, recent reports have indicated that NaVO shows inferior rate performance and significant capacity degradation during cycling due to the low electrical conductivity of V^5+^ and the pronounced electrostatic shielding effect of Zn^2+^ within the structure [12,14,25]. Therefore, defect engineering strategy enables the reduction of V^5+^ to V^4+^ in NaVO, which significantly enhances the electrochemical performance for aqueous zinc-ion battery.

In this work, we implement a defect engineering strategy through NH_4_^+^ pre-incorporation in the NaVO cathode. Partial NH_4_^+^ introduces defects and oxygen vacancies in the material, thereby improving the electrochemical performance. As a result, NaNVO delivers a high capacity of 456 mAh g^−1^ at 0.1 A g^−1^, with a capacity retention of 88% after 140 cycles and a long lifespan, maintaining 99% of its initial capacity after 2300 cycles (2 A g^−1^). Moreover, ex situ XRD and XPS demonstrate high reversibility with high structure stability for the cathode NaNVO with NH_4_^+^ pre-intercalation. This method can be applied to design high-performance cathodes.

## 2. Materials and Methods

### 2.1. Experimental Section

The synthesis of NaNVO was refined compared to previous methods [32]. Both NaNVO and NaVO were prepared using a straightforward hydrothermal approach followed by post-calcination.

### 2.2. Electrode Material Synthesis

A total of 0.3 g of polyethylene glycol (PEG-1500), 3 mmol V_2_O_5,_ and 1.5 mmol NaOH were mixed in a beaker containing 30 mL deionized water and stirred magnetically at room temperature for 0.5 h. The mixtures were then heated at 180 °C for 48 h in a 50 mL Teflon-lined autoclave. After cooling to room temperature, the products were collected and washed repeatedly with deionized water and ethanol. Subsequently, the sample was dried overnight in an oven at 60 °C to obtain the precursor, which was obtained by calcinating the collected precursor at 240 °C for 2 h under an air atmosphere at a heating rate of 2 °C min^−1^. This yields the NaVO cathode material. For NaNVO, 1.5 mmol NH_4_AC was required in the first step during the above synthesis.

### 2.3. Material Characterizations

A field emission scanning electron microscope (SEM) was obtained on JSM-7800F, JEOL Ltd. Tokyo, Japan. X-ray diffraction (XRD) pattern was recorded on a Rigaku Co., Ltd. Akishima, Japan with Cu-Kα radiation (λ = 0.15418). Fourier transform infrared (FTIR) spectroscopy was performed on a Nicolet NEXUS 670, Wisconsin, WI, USA spectrometer, and Raman spectra were acquired using an HR-800 system. X-ray photo-electron spectroscopy (XPS) was obtained on a PHI5000 Ver-saprobe III, ULVAC-PHI, Chigasaki City, Japan XPS spectrometer with an Al Ka X-ray source (1486.6 eV). Electron paramagnetic resonance (EPR) measurements were recorded on an FA-200 (JES), JEOL Ltd. Tokyo, Japan electron paramagnetic resonance spectrometer. Raman spectra were recorded on a Renishaw in Via Plus Micro-Raman spectroscopy system, London, UK equipped with a 50 mW DPSS laser at 532 nm. Rietveld refinement of the NaVO XRD patterns was performed using GSAS-I (General Structure Analysis System, Los Alamos National Laboratory) with monoclinic space group C2/m (No. 12), yielding refined structural parameters.

### 2.4. Electrochemical Measurements

The all-electrochemical measurements were carried out via stainless steel CR2032 coin-type cells. The cathodes were prepared by mixing active material (70 wt%), acetylene black (20 wt%), and polyvinylidene fluoride (PVDF) binder (10 wt%) with N-methyl-2-pyrrolidone (NMP). The average active material loading density was approximately 2.5 mg cm^−2^. For the fabrication of the zinc battery, a 12 mm zinc disc was used as the anode; meanwhile, a 3 M Zn (CF_3_SO_3_)_2_ aqueous solution (200 μL electrolyte) and glass fiber film disk (Whatman GF/D) were used as the electrolyte and separator, respectively. The homogenous mixtures were then coated on a titanium-foil Wire Mesh (SSWM) and then dried at 80 °C under vacuum for 12 h. The coin cells were assembled with Zn anode and sodium vanadate cathode in the electrolyte of 3 M Zn(CF_3_SO_3_)_2_.

Galvanostatic charge–discharge measurement was conducted by a multi-channel battery testing system (LAND CT2001A). A cyclic voltammetry (CV) test was performed using the CHI 660e electrochemical station. All the electrochemical measurements were carried out at a controlled temperature of 28 °C. The galvanostatic intermittent titration technique was carried out at a current density of 100 mA g^−1^ with a galvanostatic charge pulse of 5 min and a relaxation of 0.5 h to reach the quasi-equilibrium potential.

Basing on the galvanostatic intermittent titration technique (GITT), the diffusion coefficient of Zn^2+^ was calculated according to the following equation:

(1) $$D_{{Zn}^{2+}}=\frac{4}{\pi \tau }{\left(\frac{m_{B}V_{M}}{M_{B}S}\right)}^{2}{\left(\frac{{∆E}_{S}}{{∆E}_{\tau }}\right)}^{2}$$

where *τ* stands for the constant current pulse time, *S* is the electrode–electrolyte interface area, *m_B_*, *M_B_*, and *V_M_* are the mass, molar mass, and molar volume, respectively. *ΔE_S_* is the voltage difference for the open circuit condition, and *ΔE_τ_* is the total change in voltage during the constant current pulse.

Electrochemical impedance spectroscopy (EIS) measurements of NaVO and NaNVO electrodes were conducted using a CHI660E electrochemical workstation (CH Instruments, China) with an applied AC amplitude of 5 mV over a frequency range of 0.01 Hz to 100 kHz. The impedance spectra were recorded under open-circuit potential conditions to ensure stable interfacial characteristics, and the data acquisition conformed to Nyquist plot representation for interfacial charge transfer kinetics analysis.

Ex situ characterization was performed by disassembling coin cells under ambient conditions after electrochemical testing. The cathodes were rinsed with anhydrous ethanol to remove residual electrolyte components, followed by vacuum drying at 60 °C for 8 h prior to subsequent structural and compositional analyses.

All electrochemical tests were in the voltage range of 0.2 to 1.6 V vs. Zn^2+^/Zn.

### 2.5. Calculations of Energy Density

Energy density = Battery capacity (Ah) × Average voltage (V)/Battery weight (kg).

The battery capacity refers to the amount of charge that the battery can release. Average voltage is the average output voltage of a battery during discharging, which can be obtained by integrating the voltage data in the battery discharge curve, then dividing by the discharge time. Battery weight refers to the weight of the cathode material.

## 3. Results

**Figure 1:**
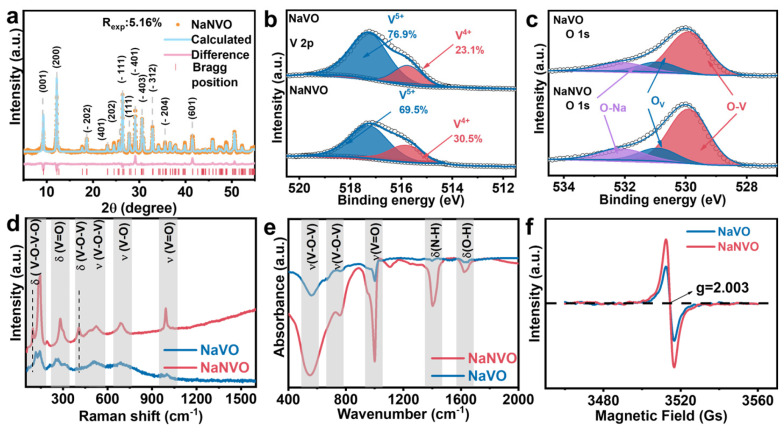
(**a**) XRD Rietveld refinement data of NaNVO. (**b**,**c**) High-resolution XPS spectra of V 2p_3/2_ and O 1s for NaVO and NaNVO. (**d**) Raman spectra. (**e**) FTIR spectra. (**f**) EPR spectra of NaVO and NaNVO.

X-ray diffraction (XRD) was employed to characterize the phase structural properties of NaNVO in Figure 1a. The products demonstrated a monoclinic crystal system structure with cell dimensions of a = 10.09 Å, b = 3.61 Å, and c = 15.39 Å. The diffraction peaks correspond well with the standard diffraction of Na_0.76_V_6_O_15_ (JCPDS No.: 75-1653) with the C2/m space group. The Rietveld refinement indicated that intercalated NH_4_^+^ did not change the crystal structure of Na_0.76_V_6_O_15_ in Figure S1. Detailed Rietveld analysis in Tables S1 and S2 shows a reduced cell volume in NaNVO with NH_4_^+^. Inductively coupled plasma optical emission spectrometry (ICP-OES) analysis verified the stoichiometric ratio of Na:V in NaVO, which is in accordance with the Na0.76V6O15 phase. This also demonstrates a significant Na-content reduction upon NH_4_^+^ incorporation (Table S3). The deconvoluted high-resolution XPS of V 2p _2/3_ reveals the reduction of V^5+^ to V^4+^ after the NH_4_^+^ intercalation, since the ratio of V^5+^ reduces from 76.9% to 69.5% and the ratio of V^4+^ increases accordingly [33]. The deconvoluted O 1s show similar O-V, O-Na, and O_v_ species for both cathodes; therefore, we can hardly distinguish a noticeable difference between NVO and NaNVO, as shown in Figure 1c. However, in Figure 1f, the g-factor of 2.003 in NaNVO possesses a stronger peak intensity than NaVO, suggesting that NaNVO possesses more oxygen vacancies than NaVO. This reveals that the decomposition of NH_4_^+^ increases the content of oxygen vacancies in NaNVO, which will benefit the reaction kinetics of NaNVO [13,14]. Both the XPS and defect analysis suggest the presence of NH_4_^+^ and the introduction of oxygen vacancies and crystal defects, which leads to better conductivity of the material and better electrochemical performance [15,20,25]. In Figure 1d, the δ(V-O-V-O) peak in NaNVO is shifted to the left compared to NaVO, which is due to the formation of structural defects [34]. Surprisingly, compared with NaVO, NaNVO shows a distinct peak at 400 cm^−1^, indicating a bridge-coordinated V-O bond, which enhances the structural stability [34,35]. Fourier transform infrared (FTIR) spectra, Figure 1e, indicated the absorption bands of the V-O bending, V-O-V, and V=O stretching vibrations, with additional bands assigned to the N-H bond at 1407.8 cm^−1^ and 1628.3 cm^−1^. This is in good accordance with the XPS, indicating the presence of NH_4_^+^ within the NaNVO structure. All the results confirm that the NH_4_^+^ intercalated NaNVO possesses more V^4+^ and oxygen vacancy compared with NVO.

**Figure 2:**
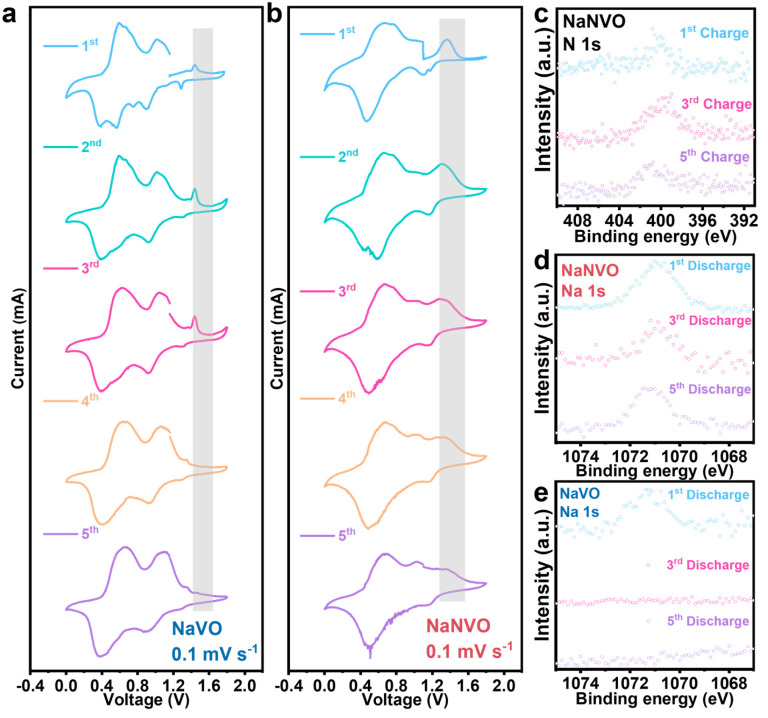
(**a**) CV curve of NaVO. (**b**) CV curve of NaNVO. (**c**) High-resolution XPS spectra for NaNVO of N 1s. (**d**) NaNVO of Na 1s. (**e**) NaVO of Na 1s.

Cyclic voltammetry (CV) tests on NaVO and NaNVO materials were shown in Figure 2a. NaVO exhibits distinct redox couples in the first cycle CV, namely Na^+^/Na, V^5+^/V^4+^, and V^4+^/V^3+^. However, as the charge–discharge cycles progress, the redox peaks corresponding to Na^+^/Na gradually diminish, indicating that Na^+^ is undergoing an irreversible process. Since the Na^+^ ions serve as the stabilizing pillar in the NaVO tunnel structure, maintaining a distance between VO_6_ units, thereby mitigating the electrostatic repulsion between Zn^2+^ and VO_6_. Consequently, the loss of Na^+^ in NaVO leads to structural instability and exacerbates the electrostatic repulsion between Zn^2+^ and VO_6_, further compromising the structural integrity of NaVO. Concurrently, the positions of V^5+^/V^4+^ and V^4+^/V^3+^ redox couples remain relatively stable. As illustrated in Figure 2b, the reduction peak of V^5+^/V^4+^ in NaNVO is elevated compared to that in NaVO, indicating that NaNVO can provide a higher voltage platform, thereby effectively enhancing the energy density. This is due to the influence of NH_4_^+^ and the presence of oxygen vacancies and defects. As the charge–discharge cycles progress, the reduction peaks of V^5+^/V^4+^ and Na^+^/Na gradually merge together. Meanwhile, the oxidation peaks of Na^+^/Na persist due to the stabilizing effect of NH_4_^+^, signifying that NaNVO achieves reversible deintercalation of Na^+^. This is crucial for maintaining the capacity and structural stability of NaNVO. To evaluate the structural stability under a prolonged electrolyte exposure, NaVO and NaNVO cathode materials (0.5 g each) were separately immersed in 9 mL electrolyte and maintained under light-protected isothermal conditions. As evidenced in Figure S2, it significantly ameliorates the dissolution of NaVO in the electrolyte.

Cyclic voltammetry analyses in Figure 2a,b suggest the different redox peak evolution during charge–discharge. The ex situ XPS at the initial five cycles confirmed that both Na^+^ and NH_4_^+^ in NaNVO show consistent appearance with the unchanged peak of N1s and Na 1s in Figure 2c,d. In contrast, the Na 1s in the NVO only showed a signal for the initial cycle and disappeared for the following cycles in Figure 2e. This can be related to the unstable structures in NVO cathode in the aqueous electrolyte. During the charge process, Na^+^ is extracted from the cathode, while both Na^+^ and Zn^2+^ ions may intercalate into the cathode during discharge. The ex situ XPS analysis of charged NaNVO electrodes confirmed the persistent presence of NH_4_^+^ species despite significant Na^+^ reduction. The ion substitution mechanism provides essential structural stabilization during electrochemical cycling. Ex situ XPS analysis of the N1s spectra in Figure 2c confirms the persistent presence of NH_4_^+^ in NaNVO, which critically facilitates the reversible Na^+^ intercalation behavior as demonstrated in Figure 2d. To elucidate the NH_4_^+^ effect on the structural stability of NaVO cathodes, comparative ex situ SEM analysis reveals that, though both NaVO and NaNVO exhibit characteristic nanorod architectures initially (Figure S3a,b), a great difference exists after the long cycle test. NaVO cathodes display as particles with visible cracks (Figure S3c), whereas the NaNVO cathode still maintains the structural integrity (Figure S3d). This pronounced morphological retention demonstrates the enhanced structural stability achieved through NH_4_^+^ in NaNVO. To further elucidate the NH_4_^+^ stabilization mechanism, CV analysis was conducted on NH_4_V_4_O_10_ cathodes under identical testing conditions (Figure S4). Crucially, NH_4_V_4_O_10_ exhibited analogous redox features to NaVO and NaNVO counterparts but notably lacked the characteristic Na^+^/Na oxidation peaks due to the absence of Na^+^ intercalation. This confirms that NH_4_^+^ remains electrochemically inert without participating in the redox reactions.

**Figure 3:**
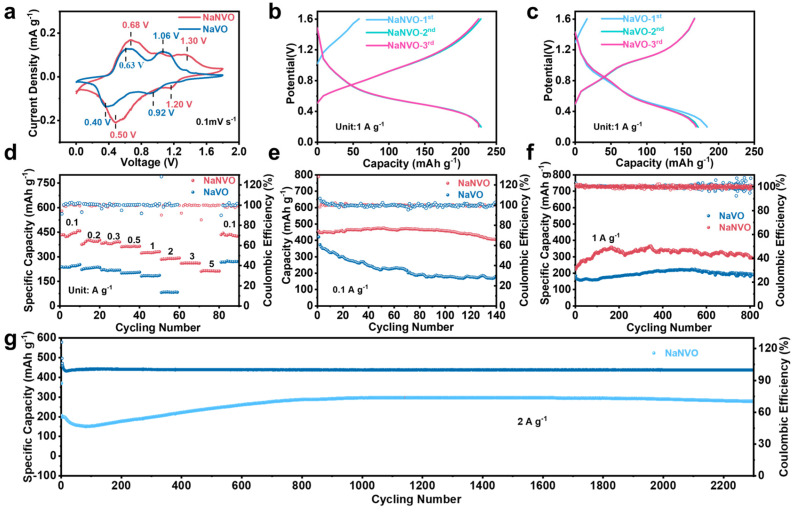
Electrochemical performance of the NaNVO and NaVO cathodes. (**a**) CV curve at a scan rate of 0.1 mV s^−1^. (**b**) GCD curves of NaNVO at a current density of 1 A g^−1^. (**c**) GCD curves of NaVO at 1 A g^−1^. (**d**) Rate capabilities at various current densities. (**e**) Long-term cycling performance at 0.1 A g^−1^. (**f**) Long-term cycling performance at 1 A g^−1^. (**g**) Long-term cycling performance at 2 A g^−1^.

The electrochemical properties were further explored in Figure 3. As demonstrated by the cyclic voltammetry (CV) analysis in Figure 3a, NaNVO exhibits a significantly enhanced discharge voltage plateau (1.3 V) compared to the pristine NaVO (1.06 V). This potential increase stems from the reversible deintercalation of Na^+^. The elevated operating voltage directly enhances the overall energy density of the full cell, suggesting promising applications in high-voltage zinc-ion battery configurations [36,37]. Figure 3b, c reveals the initial charge–discharge profiles of these two cathodes. It should be noticed that both cathodes display a low initial charge capacity, which is related to the extracted Na^+^ ions in the cathodes. The low sodium content leads to a low initial capacity and low CE. But for the following discharge, the electrodes show improved discharge capacities and CE due to the reversible Zn^2+^ intercalation/deintercalation. It reveals that NaNVO not only outperforms NaVO in terms of capacity but also in the stability. NaNVO cathodes exhibit markedly lower capacity decay in the initial cycles, indicating improved electrochemical performance and structural stability. Figure 3d depicts the initial specific capacity of NaNVO as 468 mAh g^−1^ at a current density of 0.1 A g^−1^. Notably, upon reverting to this lower current density after high-rate cycling, the capacity is fully recovered, underscoring NaNVO’s exceptional stability. Furthermore, NaNVO demonstrates remarkable capacity retention even at elevated current densities, maintaining a specific capacity of 211 mAh g^−1^ at 5 A g^−1^. Figure 3e demonstrates that NaNVO exhibits better cycle stability than NaVO at a current density of 0.1 A g^−1^. This further substantiates that the presence of NH_4_^+^ and the introduction of oxygen vacancies and crystal defects enhance the stability of the material. As demonstrated in Figure 3e, both NaNVO and NaVO cathodes exhibit impressive initial discharge capacities at 0.1 A g^−1^, but undergo pronounced capacity attenuation when cycled at elevated current densities. Figure 3f demonstrates that NaNVO maintains stable Coulombic efficiency (CE) during the cycles at 1 A g^−1^; in contrast, NaVO exhibits poor CE with low stability, potentially due to the unstable cathodes. Figure 3g further confirms the remarkable cycling stability of NaNVO at 2 A g^−1^, exhibiting an initial capacity of 302 mAh g^−1^ and retaining 99% of this capacity after 2300 cycles. Compared with other candidates, NaNVO holds a significant competitive advantage over other electrodes in Table S4.

**Figure 4:**
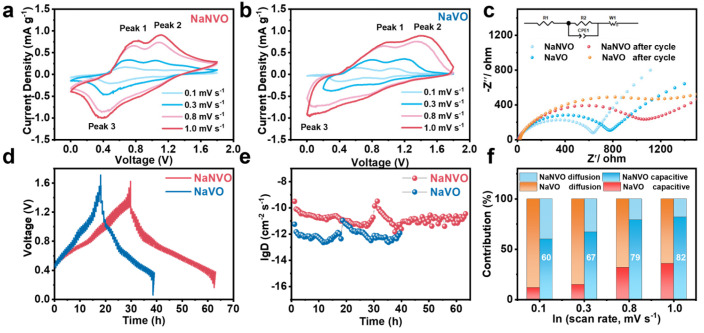
(**a**) CV curves of NaNVO at various sweep rates. (**b**) CV curves of NaVO at various sweep rates. (**c**) Nyquist plots of NaVO and NaNVO. (**d**) GITT curves. (**e**) Zn^2+^ diffusion coefficient. (**f**) Diffusion and capacitive contributions of NaVO and NNVO under different scan rates.

To further elucidate the kinetics, CV profiles of NaNVO in Figure 4a,b obtained at various sweep rates were analyzed according to the power-law, where the peak current (*i*) and scan rate (*ν*) are described as follows:

𝑖 = 𝑎𝜈^𝑏^ (2)

𝑙𝑜𝑔(𝑖) = 𝑏𝑙𝑜𝑔(𝑣) + 𝑙𝑜𝑔𝑎 (3)

where *a* and *b* are variable parameters. The *b* values can be obtained via calculating the slope of the *log (i)* versus *log (v)* plots. The *b* = 1 suggests a surface capacitive behavior, while *b* = 0.5 represents a diffusion-controlled process [38]. As the scan rate increased from 0.1 to 1.0 mV s^−1^, the *b* values of the oxidation peaks in NaNVO were 0.80 and 0.93, and it was 0.84 for the reduction peak (Figure S5). In contrast, the *b* values of the oxidation peaks in NVO were 0.54 and 0.65, and it was 0.63 for the reduction peaks (Figure S6). These results indicate that the electrochemical reactions in both NaNVO and NVO are controlled by both surface-controlled capacitance and diffusion processes.

The contribution ratio of surface capacitance to the diffusion contributions at different scan rates can be obtained from the following equations:

(4) $$i\left(v\right)=k_{1}v+k_{2}v^{1/2}$$

(5) $$\frac{i(v)}{v^{1/2}}=k_{1}v^{1/2}+k_{2}$$

The current-potential profiles were analyzed through the scan rate (v)-dependent current response *i(v)* following the power-law relationship, $i\left(v\right)=k_{1}v+k_{2}v^{1/2}$, where the linear term $k_{1}v$ quantifies surface-controlled capacitive contributions arising from combined non-Faradaic double-layer charging and surface-mediated pseudocapacitive storage, while the $k_{2}v^{1/2}$ term characterizes the diffusion-limited Faradaic process governed by semi-infinite ion transport in the bulk electrode. This deconvolution methodology enables precise discrimination between interfacial charge storage mechanisms and bulk-phase redox kinetics through quantitative parametric analysis of the voltammetric signature. The electrochemical impedance spectra (EIS) in Figure 4c reveal that NaNVO exhibits a lower charge transfer resistance (636 Ω) compared to NaVO (778 Ω) in the electrolyte, indicating enhanced interfacial charge transfer kinetics due to NH_4_^+^ modification. Consistently, GITT analysis demonstrates superior Zn^2+^ diffusivity in NaNVO, with diffusion coefficients spanning from 3.25 × 10^−10^ to 1.49 × 10^−12^ cm^2^ s^−1^ in Figure 4d, whereas NaVO shows significantly reduced values in the range from 1.09 × 10^−11^ to 2.36 × 10^−13^ cm^2^ s^−1^ in Figure 4e. This synergistic improvement in both charge transfer efficiency and ion mobility confirms the effectiveness of NH_4_^+^ incorporation in optimizing the electrode–electrolyte interfacial dynamics and bulk Zn^2+^ transport pathways. Furthermore, the capacitive contribution of NaVO reaches up to 79% at 0.8 mV s^−1^ in Figure S7, which is higher than that of NaVO in Figure 4f. The improved kinetics in NaNVO can be attributed to the oxygen vacancies, which can promote the capacitive charge storage with fast kinetics [35]. Oxygen vacancies allow fast electron transfer through defect sites, leading to rapid electron transport [13,16]. In addition, oxygen vacancies lead to a shift in the electron cloud density towards vanadium atoms, which reduces the electrostatic interactions between Zn^2+^ and the [VO_n_] layer during Zn^2+^ insertion, thus reducing the energy barrier required for Zn^2+^ intercalating, thereby promoting the diffusion kinetics [11,14,25].

**Figure 5:**
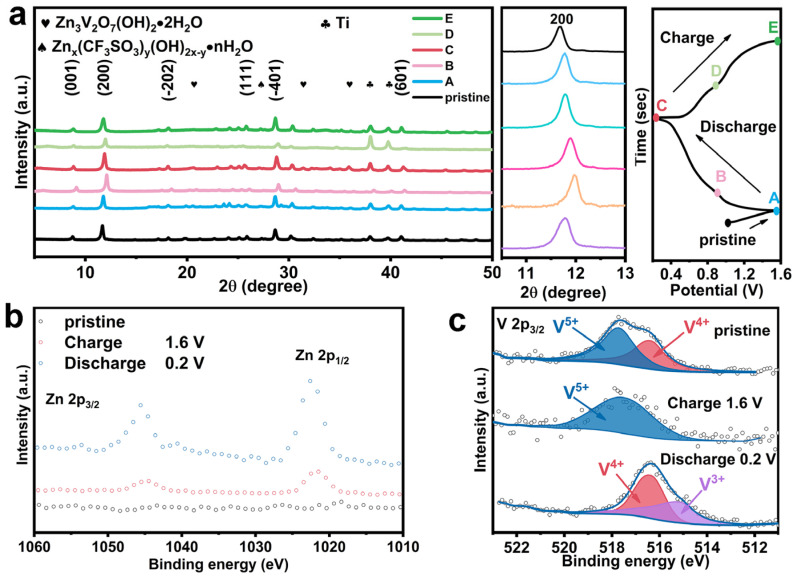
Mechanism illustrated via the ex situ measurements. (**a**) Ex situ XRD. (**b**) Ex situ XPS Zn 2p. (**c**) Ex situ XPS V 2p.

Ex situ XRD was recorded to reveal the reversibility of NANVO cathodes in Figure 5a. The typical peak (200) related to NaNVO returned to their original positions after charge–discharge, indicating the high structural reversibility in NaNVO crystal phase. Meanwhile, peaks corresponding to the side produces of Zn_x_ (CF_3_SO_3_) _y_(OH)_2x−y_·nH_2_O and Zn_3_V_2_O_7_(OH)_2_·2H_2_O appeared, implying the generation of zinc salt byproducts during the charge–discharge process. Ex situ XPS in Figure 5b further elucidated the reaction mechanism of NaNVO. The detection of Zn 2p peaks in the fully charged state implies either incomplete extraction of Zn^2+^ or the potential formation of a new phase. Figure 5c illustrates the redox processes: during the discharge, V^5+^ and V^4+^ are reduced to V^3+^ as a result of Zn^2+^ intercalation, and during the charging, V^4+^ and V^3+^ are re-oxidized to V^5+^. Therefore, the detailed electrochemical reaction process can be summarized as follows:

In the anode: *Zn*↔*Zn*^2+^ + 2*e*^−^ (6)

In the cathode: *NaNVO* + *xZn*^2+^ + 2*xe^−^* + n*H*_2_*O* ↔ *Zn_x_NaNVO*·*nH*_2_*O* (7)

NaNVO + Zn^2+^ + H_2_O + Zn (CF_3_SO_3_)_2_ ↔ Zn_x_ (CF_3_SO_3_) _y_ (OH) _2x−y_ ·nH_2_O + Zn_3_V_2_O_7_ (OH) _2_ ·2H_2_O (8)

## 4. Conclusions

In summary, NaNVO with NH_4_^+^ pre-intercalation demonstrates improved structure stability with rich oxygen vacancies and crystal defects, thereby enhancing the conductivity and stability. These modifications result in improved cyclic stability, high rate performances, and faster Zn^2+^ diffusion dynamics. Therefore, NaNVO demonstrates improved electrochemical performance, achieving a high specific capacity of 456 mAh g^−1^ at 0.1 A g^−1^. Moreover, it exhibits a capacity retention of 88% after 140 cycles and an impressive lifespan, retaining 99% of its initial capacity after 2300 cycles (2 A g^−1^). Additionally, the ex situ XRD and XPS results confirm excellent reversibility after cycling. This study suggests that NaNVO could serve as a promising candidate for next-generation energy storage devices, offering both high efficiency and long-term stability.

## Data Availability

Data are contained within the article and Supplementary Materials.

## Supplementary Materials

The following supporting information can be downloaded at https://www.mdpi.com/article/10.3390/nano15120940/s1. Figure S1: The crystal structure and the Rietveld refinement of NaVO. Figure S2: Comparison on the dissolution ability of the electrode materials in the electrolytes (a) The color change implies the stability difference between NaNVO and NVO after immerse in electrolyte over one month. (b) The concentration of vanadium dissolved in the electrolyte confirmed by the Induc-tively coupled plasma-optical emission spectrometry (ICP-OES). Figure S3: SEM of different cathode materials before cycling: (a) NaVO, (b) NaNVO. SEM of different cathode materials after 50th: (c) NaVO, (d) NaNVO. Figure S4: CV curve of NH_4_V_4_O_10_. Figure S5: The relationship between peak currents and sweep rates, and corresponding b values of NaNVO. Figure S6: The relationship between peak currents and sweep rates, and corresponding b values of NaVO. Figure S7: Capacitive contribution of the NaNVO cathode at the sweet rate of 0.8 mV s^−1^; Table S1: The cellular parameters of NaVO; Table S2: The cellular parameters of NaNVO; Table S3: The stoichiometry results for NaVO and NaNVO. Table S4: Comparison of the electrochemical properties of some vanadium-based cathode materials.
