# Supplementary material for: Stable Vacancy-Rich Sodium Vanadate as a Cathode for High-Performance Aqueous Zinc-Ion Batteries
Source: Nanomaterials (Basel). 2025 Jun 17;15(12):940. doi: 10.3390/nano15120940 (PMC12196127; doi:10.3390/nano15120940)
Supplement: Supplementary file 1 [file nanomaterials-15-00940-s001.zip › nanomaterials-3682900-supplementary.pdf]

Supporting Information

# Stable Vacancy-Rich Sodium Vanadate as a Cathode for High-Performance Aqueous Zinc-Ion Batteries

**Zhibo Xie[a #], Yongru Qu[a #], FuweiKong[a], Ruizheng Zhao[b,*], Xianfen Wang[a *]**

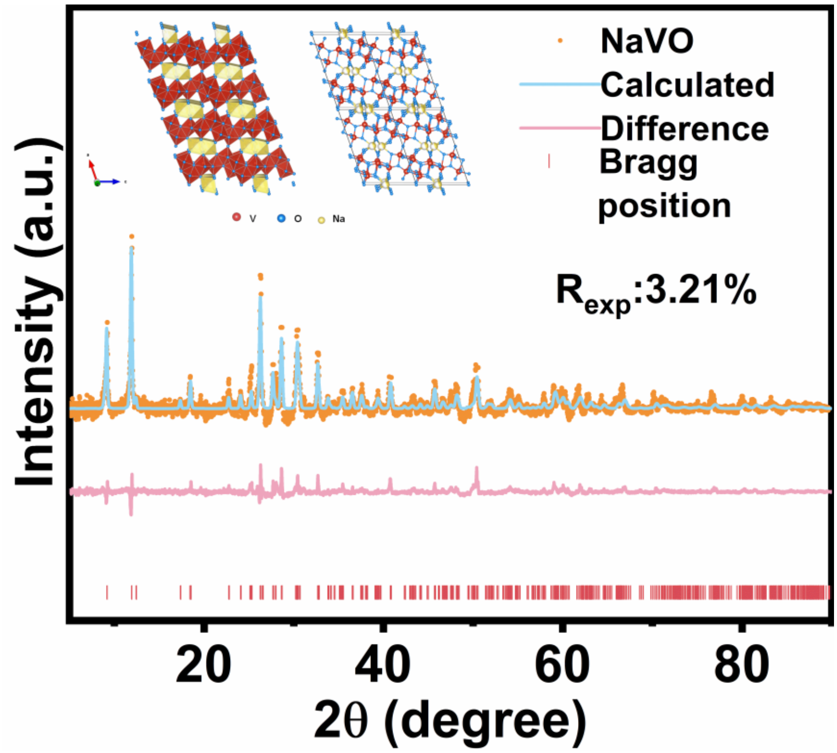


Figure S1. The crystal structure and the Rietveld refinement of NaVO.

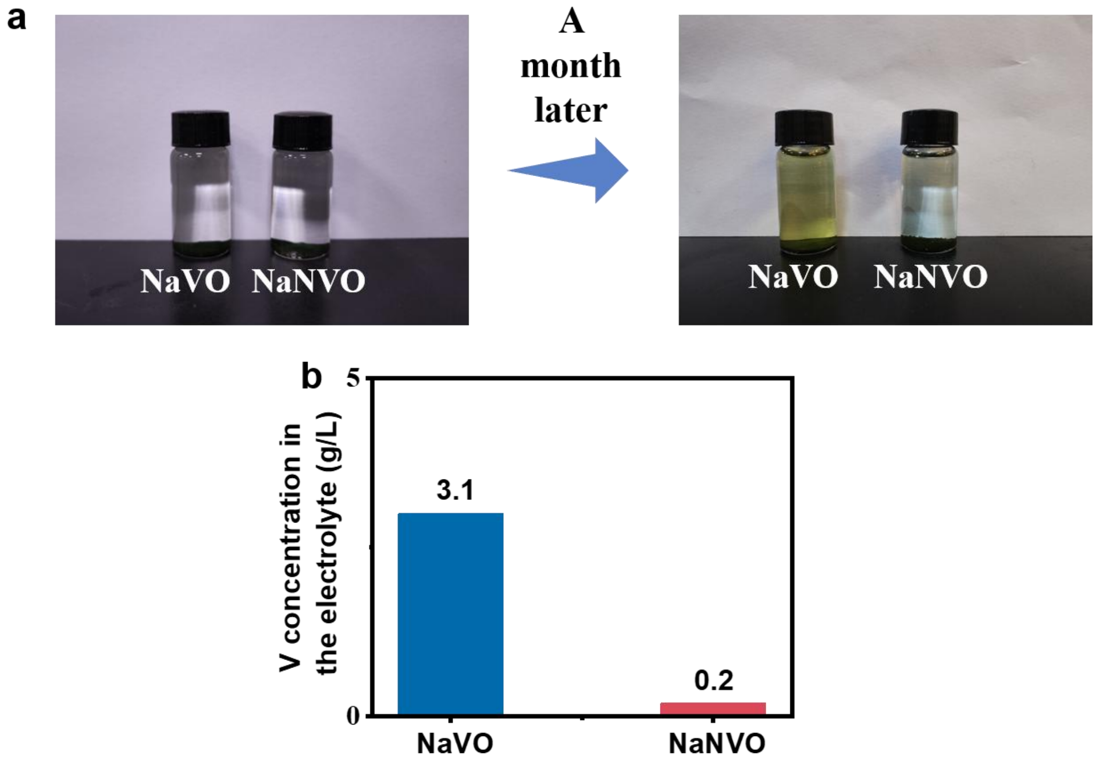


Figure S2. Comparison on the dissolution ability of the electrode materials in the electrolytes (a) The color change implies the stability difference between NaNVO and NVO after immerse in electrolyte over one month. (b) The concentration of vanadium dissolved in the electrolyte confirmed by the Induc-tively coupled plasma-optical emission spectrometry (ICP-OES).

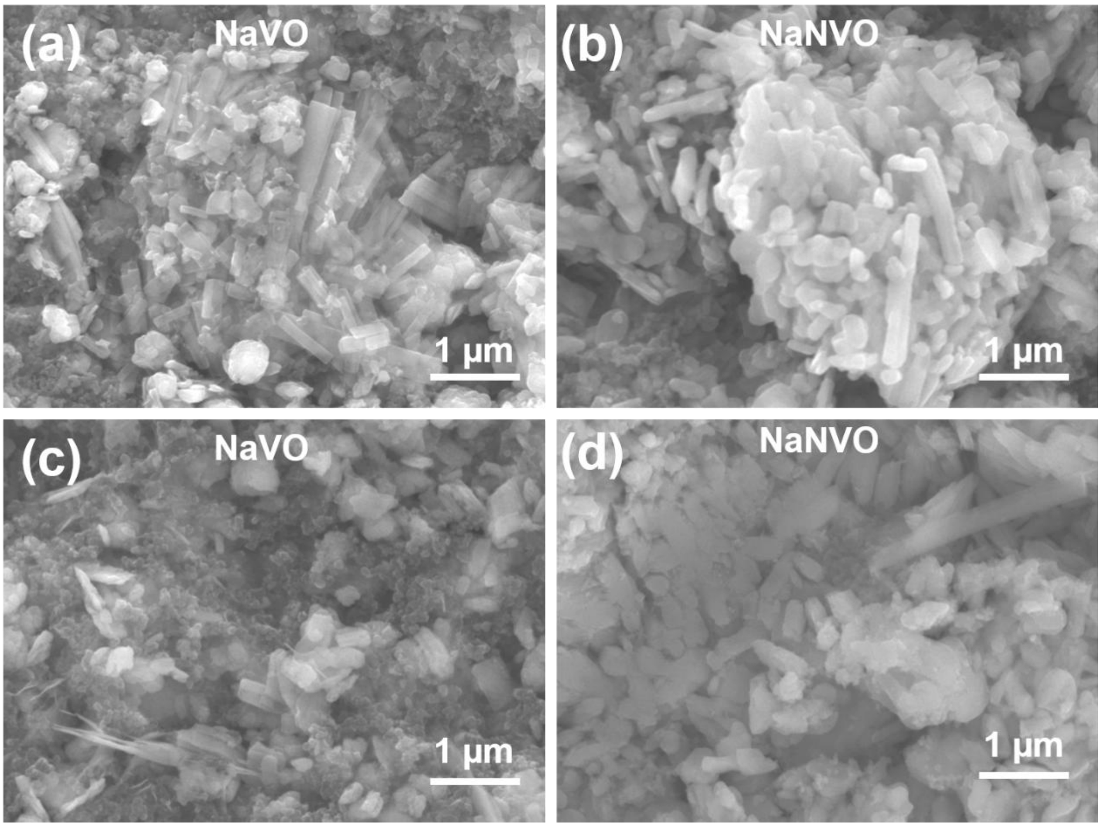


Figure S3. SEM of different cathode materials before cycling: (a) NaVO, (b) NaNVO. SEM of different cathode materials after $50^{th}$: (c) NaVO, (d) NaNVO.

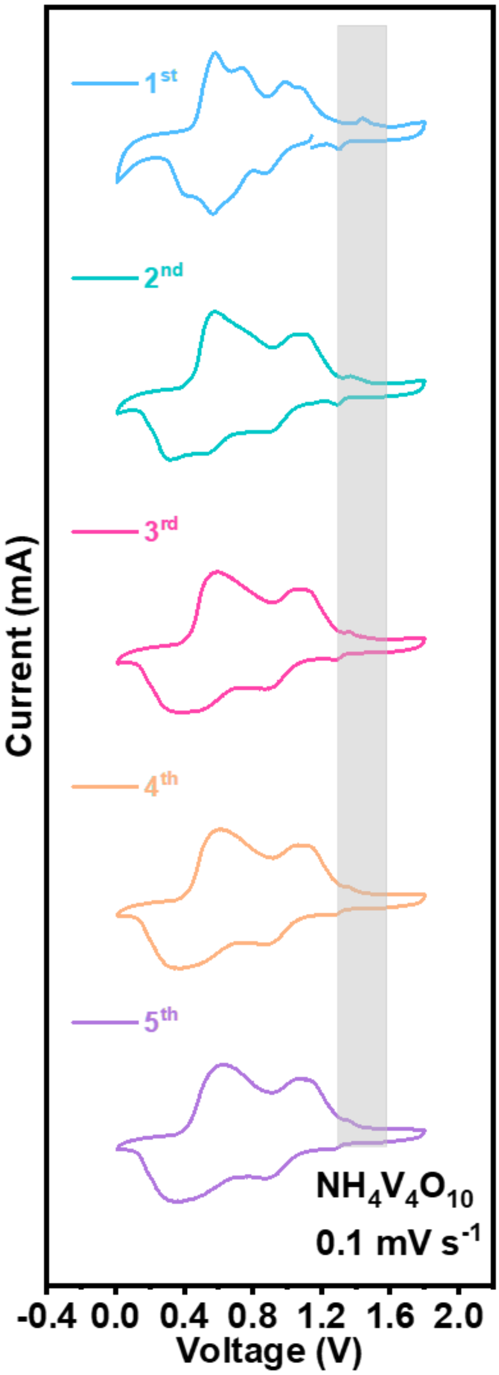


Figure S4. CV curve of $NH_4V_4O_{10}$.

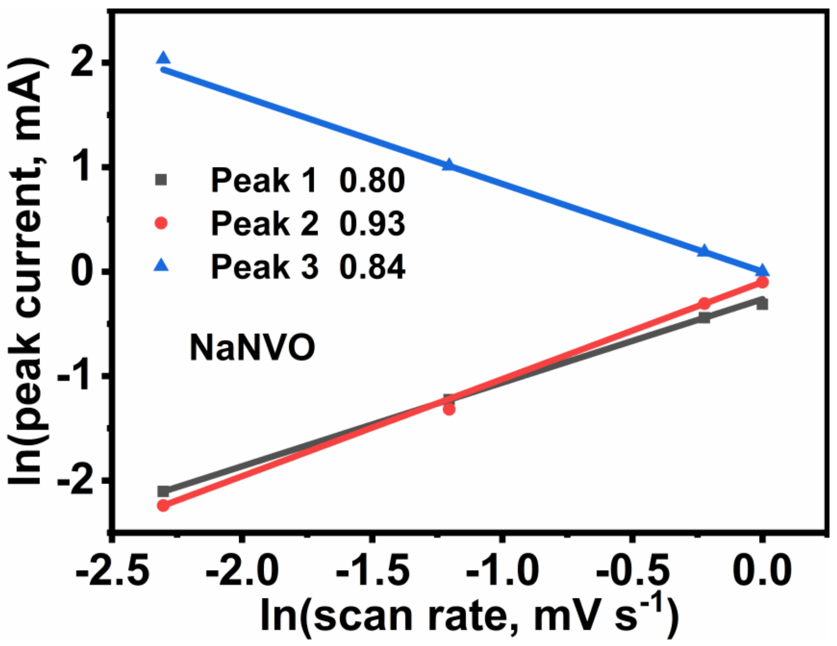


Figure. S5. The relationship between peak currents and sweep rates, and corresponding b values of NaNVO.

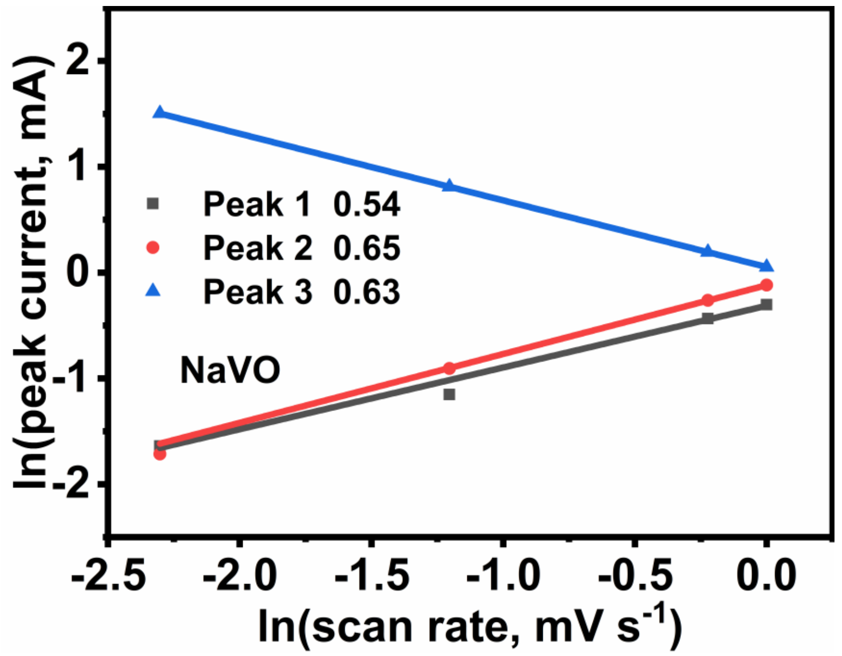


Figure S6. The relationship between peak currents and sweep rates, and corresponding b values of NaVO.

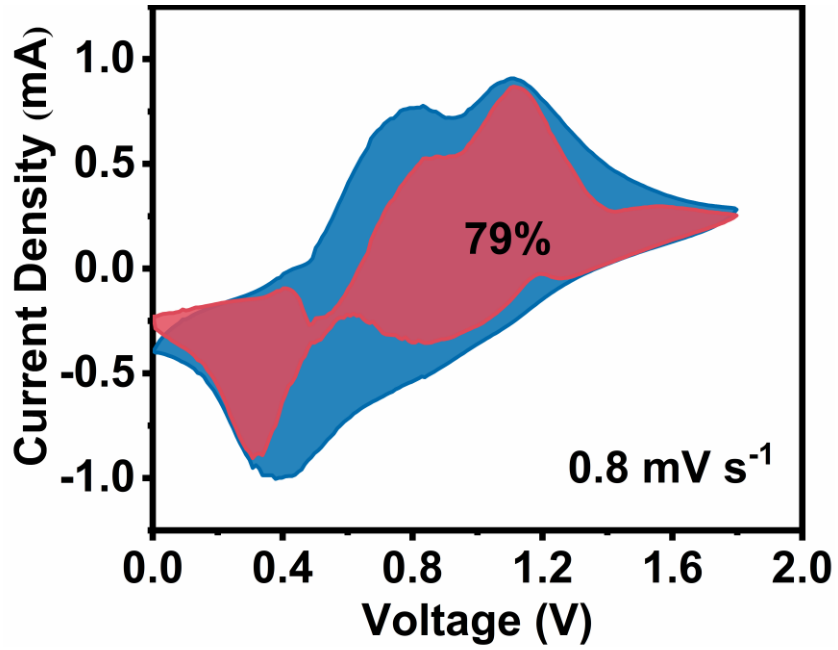


Figure S7. Capacitive contribution of the NaNVO cathode at the sweet rate of 0.8 mV $s^{-1}$

Table S1. The cellular parameters of NaVO

| Atom | Site | x | y | z | g (occupancy) |
|---|---|---|---|---|---|
| **Na** | 4i | 0.00456 | 0 | 0.40374 | 0.396 |
| **V1** | 4i | 0.33698 | 0 | 0.10205 | 0.988 |
| **V2** | 4i | 0.11566 | 0 | 0.11889 | 0.989 |
| **V3** | 4i | 0.29079 | 0 | 0.41401 | 0.988 |
| **O1** | 2a | 0 | 0 | 0 | 0.926 |
| **O2** | 4i | 0.81829 | 0 | 0.06433 | 0.932 |
| **O3** | 4i | 0.63535 | 0 | 0.0819 | 1.016 |
| **O4** | 4i | 0.43762 | 0 | 0.2244 | 1.011 |
| **O5** | 4i | 0.26598 | 0 | 0.22451 | 0.881 |
| **O6** | 4i | 0.11249 | 0 | 0.27983 | 1.006 |
| **O7** | 4i | 0.7595 | 0 | 0.42476 | 0.975 |
| **O8** | 4i | 0.40054 | 0 | 0.46892 | 1.014 |

NaVO space group: C 2/m

$a$ =10.096 Å, $b$ = 3.608 Å, $c$ = 15.583 Å, $V$ = 536.117 Å$^3$

Table S2. The cellular parameters of NaNVO

| **Atom** | **Site** | **x** | **y** | **z** | **g (occupancy)** |
|---|---|---|---|---|---|
| **Na** | 4i | -0.02202 | 0 | 0.43078 | 0.215 |
| **NH4** | 4i | -0.04442 | 0 | 0.23456 | 0.189 |
| **V1** | 4i | 0.33412 | 0 | 0.10479 | 1.019 |
| **V2** | 4i | 0.11633 | 0 | 0.11971 | 0.982 |
| **V3** | 4i | 0.28815 | 0 | 0.41837 | 0.993 |
| **O1** | 2a | 0 | 0 | 0 | 0.855 |
| **O2** | 4i | 0.8126 | 0 | 0.05509 | 0.884 |
| **O3** | 4i | 0.61949 | 0 | 0.09235 | 1.004 |
| **O4** | 4i | 0.43704 | 0 | 0.2282 | 1.006 |
| **O5** | 4i | 0.25769 | 0 | 0.21172 | 0.886 |
| **O6** | 4i | 0.11226 | 0 | 0.25678 | 1.014 |
| **O7** | 4i | 0.73442 | 0 | 0.4154 | 0.892 |
| **O8** | 4i | 0.4081 | 0 | 0.47731 | 1.022 |
| NaNVO space group: C 2/m | | | | | |
| $a$ =10.089 Å, $b$ = 3.608 Å, $c$ = 15.394 Å, $V$ = 527.989 Å$^3$ | | | | | |

Table S3. The stoichiometry results for NaVO and NaNVO.

| Samples | Measured atomic radio by ICP-OES | |
|---|---|---|
| | Na | V |
| NaVO | 0.78 | 6.05 |
| NaNVO | 0.41 | 5.98 |

.

Table S4. Comparison of the electrochemical properties of some vanadium-based cathode materials.

| Cathode material | Electrolyte | Capacit | Energy density | Capacity retention | Refs. |
|---|---|---|---|---|---|
| **$C@VO_2@V_2O_5$** | $ZnSO_4$ | 372 mAh $g^{-1}$ (0.05 A $g^{-1}$) | —— | 90.3 % after 2000 cycles at 5 A $g^{-1}$ | 1 |
| **$(NH_4)_{0.38}V_2O_5$** | $Zn(CF_3SO_3)_2$ | 465 mAh $g^{-1}$ (0.1 A $g^{-1}$) | 151.1 Wh $kg^{-1}$ | 89.3 % after 100 cycles at 0.1 A $g^{-1}$ | 2 |
| **KNVO** | $Zn(CF_3SO_3)_2$ | 464 mAh $g^{-1}$ (0.1 A $g^{-1}$) | —— | 90 % after 3000 cycles at 5 A $g^{-1}$ | 3 |
| **$Na_{0.33}V_2O_5$** | $Zn(CF_3SO_3)_2$ | 367 mAh $g^{-1}$ (0.1 A $g^{-1}$) | —— | 93% after 1000 cycles at 1 A $g^{-1}$ | 4 |
| **$NH_4V_4O_{10}$** | $Zn(CF_3SO_3)_2$ | 489 mAh $g^{-1}$ (0.5 A $g^{-1}$) | —— | 90% after 8000 cycles at 15 A $g^{-1}$ | 5 |
| **$Na_{0.58}V_2O_5·0.11H_2O$** | $Zn(CF_3SO_3)_2$ | 540 mAh $g^{-1}$ (0.2 A $g^{-1}$) | —— | 92.1% after 100 cycles at 1 A $g^{-1}$ | 6 |
| **NVO-rGO/CNT** | $Zn(CF_3SO_3)_2$ | 450 mAh $g^{-1}$ (1 A $g^{-1}$) | 280.9 Wh $kg^{-1}$ | 83.1% after 1800 cycles at 10 A $g^{-1}$ | 7 |
| **$K_{0.23}V_2O_5$** | $Zn(CF_3SO_3)_2$ | 284 mAh $g^{-1}$ (0.1 A $g^{-1}$) | —— | 92.8% after 500 cycles at 2 A $g^{-1}$ | 8 |
| **$Ca_{0.25}V_2O_5·nH_2O$** | $ZnSO_4$ | 340 mAh $g^{-1}$ (0.2 C) | 267 Wh $kg^{-1}$ | 96% after 3000 cycles at 80 C | 9 |
| **$Mg_{0.34}V_2O_5·0·.84H_2O$** | $Zn(CF_3SO_3)_2$ | 353 mAh $g^{-1}$ (0.05 A $g^{-1}$) | —— | 97% after 2000 cycles at 5 A $g^{-1}$ | 10 |
| **$Mg_{0.19}V_2O_5·0.99H_2O$** | $Zn(CF_3SO_3)_2$ | 425 mAh $g^{-1}$ (0.2 A $g^{-1}$) | 7 9 | 98.1% after 5000 cycles at 5 A $g^{-1}$ | 11 |
| **$Mg_{0.2}V_2O_5·nH_2O$** | $Zn(CF_3SO_3)_2$ | 346 mAh $g^{-1}$ (0.1 A $g^{-1}$) | —— | 83.7% after 10000 cycles at 5 A $g^{-1}$ | 12 |
| **$Zn_{0.25}V_2O_5·nH_2O$** | $ZnSO_4$ | 300 mAh $g^{-1}$ (0.3 A $g^{-1}$) | 250 Wh kg-$^{1}$ | 82% after 1000 cycles at 4.5 A $g^{-1}$ | 13 |
| **$Zn_{0.3}V_2O_5·1.5H_2O$** | $Zn(CF_3SO_3)_2$ | 426 mAh $g^{-1}$ (0.2 A $g^{-1}$) | 336 Wh $kg^{-1}$ | 96% after 20000 cycles at 10 A $g^{-1}$ | 14 |
| **$Mn_{0.26}V_2O_5·nH_2O$** | $ZnSO_4$ | 484 mAh $g^{-1}$ (0.1 A $g^{-1}$) | —— | 95% after 500 cycles at 1 A $g^{-1}$ | 15 |
| **$Ni_{0.22}V_2O_5·0.94H_2O$** | $Zn(CF_3SO_3)_2$ | 442 mAh $g^{-1}$ (0.1 A $g^{-1}$) | 287 Wh $kg^{-1}$ | 99.8% after 1500 cycles at 10 A $g^{-1}$ | 16 |
| **$Zn_{0.25}(NH_4)V_2O_5·H_2O$** | $Zn(CF_3SO_3)_2$ | 367 mAh $g^{-1}$ (0.1 A $g^{-1}$) | 244.3 W h kg $^{-1}$ | 84.6% after 1220 cycles at 1 A $g^{-1}$ | 17 |
| **$(NH_4)_{0.5}V_2O_5$** | $ZnSO_4$ | 394 mAh $g^{-1}$ (0.2 A $g^{-1}$) | —— | 91.4% after 2000 cycles at 5 A $g^{-1}$ | 18 |
| **$Al_{0.2}V_2O_5$** | $Zn(CF_3SO_3)_2$ | 448 mAh $g^{-1}$ (0.1 A $g^{-1}$) | 327.1 W h $kg^{-1}$ | 61.4% after 5000 cycles at 5 A $g^{-1}$ | 19 |
| **$NH_4V_4O_{10}$** | $Zn(CF_3SO_3)_2$ | 475 mAh $g^{-1}$ (0.4 A $g^{-1}$) | 332.25 W h $kg^{-1}$ | 90% after 2100 cycles at 5 A $g^{-1}$ | 20 |
| **NaNVO** | $Zn(CF_3SO_3)_2$ | 468 mAh $g^{-1}$ (0.1A $g^{-1}$) | 361 Wh $kg^{-1}$ | 99% after 2300 cycles at 2A $g^{-1}$ | This work |

**References**


1. Liu, H.; Hou, X.; Fang, T.; Zhang, Q.; Gong, N.; Peng, W.; Li, Y.; Zhang, F.; Fan, X. *Energy Storage Mater.* 2023, *55*, 279–288.
2. Zong, Q.; Wang, Q.; Liu, C.; Tao, D.; Wang, J.; Zhang, J.; Du, H.; Chen, J.; Zhang, Q.; Cao, G. *ACS Nano* 2022, *16*, 4588–4598.
3. Liu, M.; Li, Z.; Zhang, Y. *J. Electroanal. Chem.* 2023, *942*, 117539.
4. He, P.; Zhang, G.; Liao, X.; Yan, M.; Xu, X.; An, Q.; Liu, J.; Mai, L. *Adv. Energy Mater.* 2018, *8*, 1702463.
5. Li, S.; Xu, X.; Chen, W.; Zhao, J.; Wang, K.; Shen, J.; Chen, X.; Lu, X.; Jiao, X.; Liu, Y.; Bai, Y. *Energy Storage Mater.* 2024, *65*, 103108.
6. Pan, D.; Liu, T.; Zhang, Y.; Liu, H.; Ding, M.; Chen, L. *J. Taiwan Inst. Chem. Eng.* 2021, *127*, 276–282.
7. Xu, G.; Liu, X.; Huang, S.; Li, L.; Wei, X.; Cao, J.; Yang, L.; Chu, P. K. *ACS Appl. Mater. Interfaces* 2020, *12*, 706–716.
8. Zhang, W.; Tang, C.; Lan, B.; Chen, L.; Tang, W.; Zuo, C.; Dong, S.; An, Q.; Luo, P. *J. Alloys Compd.* 2020, *819*, 152971.
9. Xia, C.; Guo, J.; Li, P.; Zhang, X.; Alshareef, H. N. *Angew. Chem. Int. Ed.* 2018, *130*, 4007–4012.
10. Ming, F.; Liang, H.; Lei, Y.; Kandambeth, S.; Eddaoudi, M.; Alshareef, H. N. *ACS Energy Lett.* 2018, *3*, 2602–2609.
11. Zhou, W.; Chen, J.; Chen, M.; Wang, A.; Huang, A.; Xu, X.; Xu, J.; Wong, C. P. *J. Mater. Chem. A* 2020, *8*, 8397–8409.
12. Guan, J.; Shao, L.; Yu, L.; Wang, S.; Shi, X.; Cai, J.; Sun, Z. *Chem. Eng. J.* 2022, *443*, 136502.
13. Kundu, D.; Adams, B. D.; Duffort, V.; Vajargah, S. H.; Nazar, L. F. *Nat. Energy* 2016, *1*, 16119.
14. Wang, L.; Huang, K.-W.; Chen, J.; Zheng, J. *Sci. Adv.* 2019, *5*, eaax4279.
15. Xu, H.; Li, S.; Jiang, Y.; Ren, L.; Ji, M.; Shen, X. *Energy Fuels* 2021, *35*, 17948–17955.
16. Liu, Y.; Lv, J.; Cao, T.; Gong, Y.; Zhang, D. *Chem. Eng. J.* 2022, *441*, 136007.
17. Wu, J.; Yang, Z.; Chen, H.; Deng, L.; Rong, Y.; Fu, Z. *Appl. Surf. Sci.* 2022, *592*, 153137.
18. Bin, D.; Liu, Y.; Yang, B.; Huang, J.; Dong, X.; Zhang, X.; Wang, Y.; Xia, Y. *ACS Appl. Mater. Interfaces* 2019, *11*, 20796–20803.
19. Pang, Q.; He, W.; Yu, X.; Yang, S.; Zhao, H.; Fu, Y.; Xing, M.; Tian, Y.; Luo, X.; Wei, Y. *Appl. Surf. Sci.* 2021, *538*, 148043.
20. Sun, R.; Qin, Z.; Liu, X.; Wang, C.; Lu, S.; Zhang, Y.; Fan, H. *ACS Sustainable Chem. Eng.* 2021, *9*, 11769–11777.
